# The temperature and pH repertoire of the transglutaminase family is expanding

**DOI:** 10.1002/2211-5463.12839

**Published:** 2020-04-07

**Authors:** Aaron Lerner, Ajay Ramesh, Torsten Matthias

**Affiliations:** ^1^ AESKU.KIPP Institute Wendelsheim Germany

**Keywords:** food processing, microbial transglutaminase, pH, temperature, tissue transglutaminase, transglutaminase

## Abstract

Transglutaminases (TGs) play important roles in the food industry, pharmacology, and biotechnology, but as protein cross‐linkers, their complexes are stable, resistant, immunogenic, and potentially pathogenic. Many TGs have been characterized, but they operate in narrow temperature and pH range limits. In a research article in this issue, Clemens Furnes and colleagues describe a novel cold‐adapted TG from Atlantic cod, which expands the operating boundaries to a lower temperature and a wider pH. In this accompanying commentary, we discuss how this TG opens new applications in cold environments and can be deactivated by heating. New sources of TGs should be explored in hot environments like hot springs, in order to increase the temperature and widen the pH ranges for human and industrial benefits.

## Graphical abstract

Transglutaminases (TGs) play important roles in the food industry, pharmacology, and biotechnology, but their complexes are stable, resistant, immunogenic, and potentially pathogenic. Many TGs operate in narrow temperature and pH range limits. In a research article in this issue, Clemens Furnes and colleagues describe a novel cold‐adapted TG from Atlantic cod. In this accompanying commentary, we discuss how this TG opens new applications in cold environments and can be deactivated by heating.

Comments on https://doi.org/10.1002/2211-5463.12826

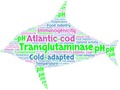

**Abbreviations**
cAcTG: cold Atlantic cod TG
CD: celiac disease
mTG: microbial transglutaminase
TG: transglutaminase
tTG: tissue transglutaminase

Transglutaminases (TGs) (http://www.chem.qmul.ac.uk/iubmb/enzyme/EC2/3/2/13.html), that is, protein‐glutamine γ‐glutamyltransferases, are pleiotropic, enigmatic, and multifunctional enzymes expressed extensively and ubiquitously in prokaryotes and eukaryotes. Their biological functions span all mammalian tissues, invertebrates, plants, fungi, yeasts, and microbial cells. Even viruses have been described to possess TG‐like activity. They represent a suprafamily, and in human, nine members of TGs have been described, playing a crucial role in homeostasis and in pathological disorders [1]. They catalyze the formation of a covalent isopeptide bond, cross‐linking a free amine group (acyl acceptor) and the γ‐carboxamide group of protein or peptide‐bound glutamine (acyl donor), resulting in post‐translational modification of proteins/peptides [2]. Their protein cross‐linking or deamidation capacities are the two main mechanisms by which they exert their functions. Interestingly, they possess additional enzymatic activities, such as GTP‐dependent signal transduction, isomerase, and ATP‐dependent kinase [3]. Microbial TG (mTG) is a member of the TG family, and despite poor sequence homology, it functionally imitates the human tissue TG (tTG), which is the autoantigen of celiac disease (CD) [4]. mTG is a food additive, heavily used in the processed food industry as a universal cross‐linker, and nicknamed ‘meat glue’. Despite the manufacturer's claims of being safe and it being categorized as GRAS (generally recognized as safe), its cross‐linked gliadin complexes were recently shown to be immunogenic and potentially pathogenic in CD [5, 6, 7]. This was a topic of criticism as it was assumed that no active mTG reached the human intestinal lumen, due to its industrial temperature inactivation and inability to resist the gastric acidic pH [8]. In this regard, Alvarez et al. should be congratulated for addressing the topic of temperature and pH dependency of TG, by characterizing a novel cold‐adapted TG enzyme and indicating its potential application for medicine and food processing, as described in a research article published in this issue [9]. In the present accompanying commentary, we expand discussion from the animal TG repertoire to human enteric lumen TG activity. More specifically, we discuss the features of cold Atlantic cod TG (cAcTG) temperature and pH dependency with relation to the need of processed food manufacturers for more adapted TGs that will operate under more extreme temperatures and pH environments.

## Human gut lumen sources of transglutaminases

Endogenous tTG is localized in the gut epithelial lining, but gut luminal mTG cargo originates from extra‐ and intra‐intestinal sources, as shown in Table 1.

**Table 1:** Enteric luminal sources of mTG (adapted from Refs. [2, 6, 10, 11]).

Extra‐intestinal	Intra‐intestinal
Processed food additive	Microbiome
Pathobionts	Dysbiome
Probiotics	Yeasts
Plants	Fungi
Vegetables	Viruses
Meat	

## The importance of temperature and pH dependency of transglutaminases for the processed food industry

According to the manufacturers of industrial mTG and critics in the literature [8, 12], the enzyme is deactivated/destroyed during heating of processed food and cannot survive the acidic gastric pH, thus contradicting its immunogenic and pathogenic capacities in CD.

It was lately shown that CD patients mount specific antibodies to the cross‐linked mTG–gliadin complexes and not to the mTG enzyme itself [5, 6, 7, 10, 12]. Secondly, it is known that those covalently linked complexes are resistant to proteases, detergents, bile acids, and a wide range of pH. Thirdly, when heated, they become even more immunogenic [12, 13]. More so, they are created ex vivo, during the industrial processing procedures, and are thus consumed as such. Lastly, there are substantial enteric mTG activity and gliadin peptides in the lumen to cross‐link them in situ. Very intriguing is Stricker *et al*.'s [13] observations that mTG and gliadin molecules are internalized through human enterocytes to lodge below the epithelium and thus face the mucosal immune systems.

Regarding the temperature dependency and sensitivity to high industrial or home cooking, heating increases the immunogenicity of the complexes and many industrial processes do not use high temperatures, for example, raw fish and meat, salads, and sauces. Most likely, more epitopes are exteriorized during heat denaturation and stimulate the immune system to react. If heated to 60–65 °C, mTG–gliadin complexes are created and can potentially reach the human lumen after being consumed (Ramesh Ajay, personal communication) [12, 13].

Several arguments stand against the criticism that mTG is deactivated during gastric passage [8, 12].

According to our preliminary results (Ramesh A, personal unpublished communication), mTG is active and cross‐links gliadin molecules at pH 4.0 and above. By screening gastric physiology, several facts are apparent: (a) Infants and the elderly have a higher gastric pH. (b) The stomach topographical distribution of pH is not uniform. Some areas are less acidic with pH > 4. (c) During meals and during the immediate postprandial period, the acidic pH is neutralized. (d) Alkaline reflux is increasingly detected by pH‐metry and impedance tests, and (6) acid suppression and proton pump inhibitor consumption are very prevalent, thereby increasing gastric pH > 4. In fact, when supermarket shelves' meat and meat products were analyzed, many were found to contain TG [14].

The reader might ask how all of the above is related to the cAcTG? [9]. It is related to the heavy use of mTG in processed food with the immunogenicity and potential pathogenicity of its cross‐linked protein complexes [2, 5, 6, 7, 10, 12, 13, 14]. One wonders if by incorporating different TG genes with different temperature and pH range optimal activities into microbes, the actual detrimental *Streptomyces mobaraensis*‐originated mTG will be cross‐linked in acidic and more extreme thermal environments. The newly identified cAcTG may be a candidate for colder reactions, and thermostable mTG can be used for heated/boiled/cooked food product manufacturing. While TG isolated from cold Atlantic cod fish has mammalian features, such as the need for calcium for activation [9], thermophilic mTG, to our knowledge, was not previously described. Active thermophilic bacteria from hot springs are constantly reported. In a recent report from Turkey, 85 bacteria were isolated, sequenced, and characterized. Active amylase, lipase, and protease were detected, but, unfortunately, TG was not studied [15]. On a second thought, mTG, which is a survival factor for the bacteria that have unwanted side effects on human immune and physical protective barriers, intestinal permeability, and mucus quality [2, 5, 6, 7, 10, 12], can be replaced for food processing, by a more friendly mammalian or other eukaryotic TG. If isolated and characterized from cold and hot habitats, the food industry might gain a friendlier and safer TG with a wider temperature and pH range of activity.

## Summary

Characterizing and sequencing the cAcTG [9] represents a new potential strategy for molecular stable, resistant covalent cross‐linking in cold milieu, not only in the processed food facilities, but also in pharmacology, biotechnology, bioconjugation, and antibody–drug conjugates for diagnostic laboratories and medical therapy. The cold‐adapted enzyme expends TG family versatility, maximizes TG advantages, widens their temperature and pH repertoire of activity, and might avoid undesired cross‐linked products. We hope that the present commentary will encourage the scientific community to explore TG activities in thermophilic bacteria residing in hot springs.

## Conflict of interest

The authors declare no conflict of interest.

## Author contributions

LA wrote the manuscript. RA screened the literature. MT designed, edited, and revised the manuscript.
